# Venoms and Drugs: Advancing Venom-Based Therapeutics from Molecular Insights to Translational Applications

**DOI:** 10.3390/toxins18100411

**Published:** 2026-09-24

**Authors:** Euikyung Kim

**Affiliations:** Institute of Animal Medicine, College of Veterinary Medicine, Gyeongsang National University, Jinju 52828, Republic of Korea; ekim@gnu.ac.kr

## 1. Introduction

Animal venoms are among nature’s most sophisticated biochemical systems. Evolved under strong ecological selection for predation, defense, and competition, they contain complex mixtures of peptides, proteins, enzymes, and small molecules that rapidly perturb essential physiological processes. Their targets include ion channels, membrane receptors, enzymes, coagulation factors, cellular membranes, and immune and inflammatory pathways. Repeated recruitment and diversification of toxin families across phylogenetically distant lineages have generated an exceptionally broad repertoire of molecular structures and biological activities [1,2,3].

The potency and target selectivity of venom components make them valuable not only for understanding envenoming, but also as molecular probes and starting points for drug discovery. Venom-derived peptides and proteins can reveal physiological pathways, validate pharmacological targets, and provide structurally constrained scaffolds for therapeutic design. Toxinology has therefore expanded from descriptive venom characterization into a multidisciplinary field encompassing pharmacology, medicinal chemistry, structural biology, biotechnology, and translational medicine [4,5,6,7].

The therapeutic value of this field is demonstrated by several medicines developed directly or indirectly from animal toxins, including captopril, eptifibatide, tirofiban, ziconotide, exenatide, and lixisenatide [8,9,10]. These examples show that evolutionarily optimized molecules can be transformed into clinically useful agents through rational modification, synthetic simplification, or formulation development. Nevertheless, approved toxin-inspired drugs remain few relative to the molecular diversity of venoms. Limited stability, rapid degradation, unfavorable pharmacokinetics, immunogenicity, systemic or off-target toxicity, restricted administration routes, and manufacturing complexity continue to impede translation [8,9,10,11,12].

Technological advances are beginning to address these constraints. Integrative venomics combines proteomic, transcriptomic, genomic, and increasingly metabolomic approaches to identify and compare venom components at unprecedented depth [13,14]. Mechanistic studies of toxin families have clarified interactions with physiologically important targets and provided templates for hemostatic and antithrombotic drug development [15]. In parallel, molecular docking, virtual screening, quantitative structure–activity analysis, and molecular dynamics simulations support the investigation of toxin–target recognition and the prioritization of candidates for experimental testing [16,17,18]. Artificial intelligence-based structure-prediction systems, exemplified by AlphaFold and AlphaFold 3, further expand opportunities to model toxin structures and biomolecular complexes [19,20,21]. Such predictions remain hypotheses requiring experimental validation, particularly when activity depends on conformational dynamics, membrane environments, post-translational modifications, or unusual disulfide connectivity. Together with peptide engineering and delivery technologies, these methods can help convert natural toxins into more selective, stable, and clinically practical candidates [10,12,22].

The biomedical relevance of venom science also extends beyond drug discovery. Snakebite envenoming remains a major clinical and public-health problem, especially in resource-limited regions where effective antivenoms may be unavailable or inadequately matched to local venoms [23,24,25]. Conversely, controlled exposure to venom allergens through Hymenoptera venom immunotherapy is an established intervention that provides substantial protection against severe systemic sting reactions [26,27]. Contemporary venom research thus spans a broad translational continuum, from improving the treatment of envenoming and venom allergy to exploiting toxin molecules as pharmacological tools and therapeutic leads. Within this continuum, the boundary between toxin and medicine is determined not only by intrinsic molecular activity, but also by dose, selectivity, delivery, engineering, and clinical context.

## 2. Rationale and Scope of the Special Issue

The Special Issue *Venoms and Drugs* was conceived to examine the interface between the biological activities of animal venoms and their pharmaceutical, biotechnological, and clinical applications. Its premise was that therapeutic exploitation requires more than the identification of potent bioactivity; it also requires an understanding of molecular structure, biological target, mechanism of action, therapeutic index, formulation, and translational feasibility. The issue therefore welcomed original research and reviews addressing venom-component characterization, toxin–target interactions, molecular mechanisms, venom-derived peptides and proteins, bioengineered analogues, preclinical and clinical evaluation, and innovative strategies for venom-based product development.

The ten published contributions illustrate this breadth. Biodiscovery studies examined ACE-inhibitory peptides from jellyfish venom, anthelmintic activity in stonefish ichthyocrinotoxins, immunomodulatory effects of amphibian skin secretions, and antimicrobial applications of coral-snake venom L-amino acid oxidase [28,29,30,31]. Mechanistic and structural studies investigated mitochondrial and immunological effects of *Bothrops diporus* phospholipases A2, the anticoagulant properties of crotoxin and related phospholipases, and the membrane-associated behavior of the wasp-venom allergen Ves a 1 [32,33,34]. Clinical and public-health contributions addressed long-term outcomes of Hymenoptera venom immunotherapy and independent preclinical evaluation of antivenoms against medically important Kenyan snakes [35,36]. Finally, electrical cell-substrate impedance sensing was evaluated as a real-time in vitro platform for studying venom-induced cellular injury and direct toxin inhibitors [37].

Collectively, these articles extend the concept of “venoms and drugs” beyond the search for isolated therapeutic molecules. They encompass natural-product discovery, mechanistic pharmacology, computational modeling, biomaterial development, therapeutic optimization, antivenom quality assurance, immunotherapy, and advanced preclinical methodology. The following overview groups the contributions by their principal scientific themes and considers their collective significance for venom-inspired therapeutics and translational toxinology.

## 3. Overview of the Contributions

The contributions span terrestrial and marine organisms and applications ranging from cardiovascular and antiparasitic lead discovery to immunomodulation, antimicrobial biomaterials, antivenom quality control, and venom immunotherapy. Despite their diversity, they share a common progression from biological discovery to mechanistic characterization and translational evaluation. Table 1 summarizes their principal findings and potential applications.

### 3.1. Biodiversity-Driven Discovery of Bioactive Molecules and Therapeutic Leads

Prakash et al. investigated a hydrolysate of *Nemopilema nomurai* jellyfish venom as a source of angiotensin-converting enzyme (ACE)-inhibitory peptides [28]. Bioactivity-guided fractionation identified a highly active chromatographic fraction, and tandem mass spectrometry revealed the sequences IVGRPLANG and IGDEPRHQYL; docking analyses supported plausible interactions with ACE. The work illustrates how controlled proteolysis can release smaller pharmacologically relevant peptides from complex venom proteins. However, synthesis and direct testing of the individual peptides, followed by stability, pharmacokinetic, efficacy, and safety studies, will be required to establish their value as antihypertensive leads [4,8,10,12,22].

Lennox-Bulow et al. examined ichthyocrinotoxins secreted by *Synanceia horrida* and *S. verrucosa* as potential anthelmintics [29]. Both secretions impaired the motility of infective *Nippostrongylus brasiliensis* larvae. Fractionation revealed distinct activity profiles: activity in *S. verrucosa* appeared to involve cooperation between higher- and lower-molecular-mass components, whereas the fraction below 3 kDa from *S. horrida* retained substantial activity with limited cytotoxicity in the tested mammalian cells. These findings identify an underexplored marine source of antiparasitic leads while emphasizing the need to separate useful activity from the nonspecific toxicity of crude secretions.

Oliveira et al. screened cutaneous secretions from a broad panel of amphibian species in human chondrocytes, synoviocytes, and macrophages relevant to joint inflammation [30]. The secretions produced marked species-dependent differences in cytotoxicity and cytokine modulation. Some were pro-inflammatory, whereas selected secretions reduced inflammatory cytokine production under defined conditions. Rather than yielding a single immediately translatable candidate, the study provides a comparative biological map for subsequent activity-guided isolation and illustrates why concentration, cytotoxicity, and cellular context must be considered when interpreting complex defensive secretions.

Núñez Rangel et al. evaluated *Micrurus* venoms and purified L-amino acid oxidase (LAAO) against phytopathogenic bacteria and fungi [31]. Venoms from *M. ancoralis*, *M. mipartitus*, and *M. dumerilii* inhibited relevant *Xanthomonas* and *Fusarium* strains, with LAAO identified as a major active component. Incorporation of venom or purified LAAO into gelatin films produced antimicrobial materials that extended strawberry shelf life without substantially altering key fruit properties. This contribution broadens venom translation beyond human therapeutics to include sustainable biocontrol and active food packaging.

Together, these studies emphasize biodiversity as a source of structurally and functionally distinct molecules [1,2,3,7]. They also show that activity in a crude venom or secretion is only the beginning of development: purification, molecular identification, mechanism, selectivity, safety, formulation, and scalable production must be addressed before a biological effect becomes a practical product [8,10,11,12,22].

### 3.2. Mechanistic and Structural Insights into Venom Components

Sasovsky et al. examined PLA2s from *Bothrops diporus* venom using biochemical, bioenergetic, cellular, and cytokine analyses [32]. In MDA-MB-231 breast cancer cells, pooled PLA2s reduced metabolic activity and proliferation while inducing mitochondrial reactive oxygen species, membrane-potential hyperpolarization, and changes in NADH autofluorescence, consistent with sustained mitochondrial stress. In peripheral blood mononuclear cells, the preparations generated temporally dynamic cytokine responses, and an individual PLA2 fraction showed a distinct immunomodulatory profile. The study therefore links tumor-cell bioenergetics with isoform-dependent immune effects and cautions against treating PLA2s as a functionally uniform toxin family.

The review by Brasileiro-Martins et al. examines anticoagulant snake-venom PLA2s, with emphasis on crotoxin from *Crotalus durissus* [33]. The authors integrate evidence for anticoagulation through hydrolysis of procoagulant phospholipids, disruption of coagulation-complex assembly, and modulation of inflammatory and endothelial pathways. This analysis complements broader knowledge of anticoagulant venom proteins [15] and highlights crotoxin as a mechanistic template for antithrombotic development, while recognizing that neurotoxicity, myotoxicity, and systemic effects necessitate molecular dissection and rational modification.

Pattaranggoon et al. used all-atom molecular dynamics to investigate Ves a 1, a phospholipase allergen from *Vespa affinis*, in a lipid-bilayer environment and in the presence of voxilaprevir [34]. The simulations implicated lid regions, the catalytic triad, and an auxiliary site in dynamic membrane interactions and suggested ligand-induced changes near the catalytic region. The study demonstrates that toxin behavior may depend on membrane composition and conformational dynamics that cannot be inferred from a static structure alone [16,17,18]. AI-based prediction can accelerate model generation [19,20,21], but dynamic simulation and experimental validation remain essential.

Collectively, these contributions examine venom pharmacology across scales, from mitochondrial and cytokine networks to coagulation pathways and atomistic membrane interactions. Such multiscale analysis is central to distinguishing therapeutically useful target engagement from secondary or nonspecific toxicity.

### 3.3. Clinical and Public-Health Translation of Venom Research

Panzera et al. reported five-year real-world outcomes of Hymenoptera venom immunotherapy in 35 patients with previous severe systemic sting reactions [35]. All patients tolerated maintenance therapy, and none of the 17 individuals who experienced accidental re-stings developed another severe systemic reaction. Longitudinal serology showed significant reductions in several immunological parameters, with variation according to the relevant insect venom. The findings support the long-term protective and disease-modifying role of venom immunotherapy and complement current guideline-based evidence [26,27].

Musabyimana et al. assessed four antivenoms against venoms from five medically important Kenyan snake species using immunological binding assays and murine neutralization experiments [36]. SAIMR polyvalent, AFRIVEN, and PANAF-Premium showed broad reactivity and substantial neutralizing potency, whereas Inoserp had lower immunoreactivity and failed to neutralize several venoms at manufacturer-claimed doses. The study demonstrates that product labeling cannot substitute for independent, geographically relevant efficacy testing and provides a direct evidence base for national quality control, regulatory oversight, and procurement decisions [14,23,25].

These studies illustrate two complementary clinical uses of venom-related interventions. Venom immunotherapy uses standardized exposure to induce protective immune tolerance, whereas antivenom therapy uses exogenous antibodies to neutralize circulating toxins. Both require accurate biological characterization, standardized products, clinically meaningful outcomes, and sustained quality assurance.

### 3.4. Innovative Preclinical Platforms for Therapeutic Evaluation

Choudhury et al. evaluated electrical cell-substrate impedance sensing (ECIS) as a real-time method for monitoring cellular responses to three viper and three elapid venoms, alone and in combination with the PLA2 inhibitor varespladib and the metalloproteinase inhibitor marimastat [37]. Parallel coagulation testing distinguished effects on cellular injury from effects on coagulotoxicity. The results showed that inhibitor performance depends on both venom composition and the pathological endpoint assessed; protection against one effect does not necessarily imply protection against another.

ECIS captures the onset and progression of cellular injury more continuously than conventional endpoint assays and offers a scalable approach for screening venoms, toxin fractions, and candidate inhibitors before confirmatory animal studies. Although it cannot reproduce the full vascular, immune, neuromuscular, and systemic complexity of envenoming, it advances quantitative preclinical testing while supporting replacement, reduction, and refinement of animal use [6,8,17,38].

### 3.5. Collective Significance of the Special Issue

Across the ten contributions, several principles emerge. Venom research remains dependent on biodiversity, including organisms and secretions that have received limited pharmacological attention. Therapeutic value cannot be judged by potency alone; molecular identity, selectivity, mechanism, toxicity, formulation, and manufacturability must be considered together. Computational and advanced in vitro approaches increasingly complement biochemical and in vivo methods, while clinical translation requires attention to regional biological variation, product quality, regulatory standards, and real-world outcomes.

The Special Issue therefore presents venom-derived innovation as a continuum. At one end lies screening of complex natural secretions; at the other are clinically established interventions such as venom immunotherapy and regionally validated antivenoms. Between these endpoints are purification, structural analysis, mechanistic pharmacology, computational modeling, safety assessment, formulation, and preclinical validation. Progress will depend on integrating these stages rather than relying on any single technology or biological activity.

## 4. Challenges and Future Perspectives

The principal translational challenge is that the properties that make toxins effective in predation or defense—high potency, rapid action, and disruption of essential physiology—may also produce narrow therapeutic windows, systemic toxicity, or irreversible tissue injury. A toxin acting strongly at a relevant target is not a viable drug lead until selectivity, exposure–response relationships, reversibility, and safety margins are established [5,6,8,9,10,11,12]. Venom peptides and proteins also face enzymatic instability, short circulating half-lives, limited oral bioavailability, immunogenicity, tissue-penetration barriers, and manufacturing complexity [8,10,11,12,22]. Rational sequence modification, truncation, cyclization, non-natural amino acids, conjugation, and targeted delivery may address these liabilities. In many cases, the optimal product will not be the native toxin, but a minimized peptide, engineered protein, or small molecule that retains the desired mechanism while removing determinants of toxicity [4,5,8,10,12,22].

Venom complexity and variability present a second challenge. Composition can differ among species, populations, geographic regions, developmental stages, sexes, diets, and ecological conditions [1,2,3,39]. Crude activity may arise from one dominant toxin, additive effects, or synergy. The fraction-dependent activity of stonefish ichthyocrinotoxins, the heterogeneous effects of amphibian secretions, and identification of LAAO as a major antimicrobial component of *Micrurus* venom illustrate why activity-guided fractionation should be integrated with transcriptomics, proteomics, and quantitative venomics [13,14,29,30,31].

Greater mechanistic resolution is equally important. Venom components may act through catalysis, receptor binding, membrane disruption, or indirect regulation of inflammatory and metabolic pathways. The *B. diporus* PLA2 study shows that mitochondrial stress and immune signaling can occur simultaneously and differ among isoforms [32], while crotoxin affects hemostasis through both phospholipid hydrolysis and broader coagulation-inflammatory interactions [15,33]. Future work should combine target engagement, pathway analysis, temporal cellular responses, and organism-level pharmacology to distinguish primary mechanisms from secondary toxicity.

Structural and computational methods will increasingly support this process. Docking and molecular dynamics can help identify residues associated with activity or toxicity and guide analogue design [16,17,18]. The Ves a 1 study demonstrates that membranes and protein dynamics can substantially influence inhibitor binding [34]. AlphaFold and AlphaFold 3 can accelerate prediction of toxin structures and interactions [19,20,21], but confidence scores must not be equated with validated pharmacology. Unusual disulfide patterns, flexible loops, post-translational modifications, oligomeric interfaces, and membrane-dependent conformations remain challenging. Computational results should therefore be treated as testable hypotheses and integrated with biochemical, biophysical, cellular, and in vivo evidence.

The usefulness of artificial intelligence will also depend on data quality. Available datasets are enriched for well-studied toxin families, whereas inactive analogs, negative results, complete dose–response data, toxicity endpoints, and pharmacokinetic observations are reported less consistently. Future resources should link sequence and structural data to standardized activity, selectivity, toxicity, and experimental metadata. Prospective evaluation on independent toxins and clearly defined applicability domains will be necessary before predictive models can reliably prioritize therapeutic candidates [13,17,19,20,40].

Preclinical testing must become more predictive, reproducible, and ethically sustainable. The ECIS platform offers real-time measurement of cellular injury and inhibitor-mediated protection and can be paired with coagulation assays to capture distinct pathological endpoints [37]. Future systems should incorporate human primary cells, organoids, microphysiological models, vascular and immune co-cultures, and clinically relevant exposure schedules [38]. These approaches will not immediately replace animal studies, but they can improve candidate selection, clarify mechanisms, and reduce unnecessary animal use.

For envenoming and venom allergy, translation also depends on regional relevance and long-term clinical evidence. The Kenyan study shows that immunological binding alone does not guarantee neutralization and that efficacy must be tested against locally important venoms [14,23,25,36]. Independent national or regional quality-control capacity can therefore strengthen procurement and patient safety. Likewise, five-year outcomes of venom immunotherapy demonstrate the value of real-world follow-up [26,27,35]; larger multicenter cohorts and harmonized outcomes could help identify predictors of protection, relapse, and adverse reactions.

Ultimately, venom-based innovation requires collaboration among toxinologists, pharmacologists, structural biologists, computational scientists, clinicians, engineers, manufacturers, and regulators. A productive pathway should begin with ethically sourced, taxonomically validated material and proceed through molecular identification, mechanism-based screening, structural analysis, safety assessment, formulation, scalable production, and clinically relevant validation [28,29,30,31,32,33,34,35,36,37]. Integrated in this way, venoms can be approached not merely as toxic mixtures but as evolutionarily optimized molecular libraries whose beneficial activities may be separated from harmful effects and developed into therapeutic and biotechnological solutions.

## Figures and Tables

**Table 1:** Summary of the contributions published in the Special Issue *Venoms and Drugs*.

Ref.	Biological Source	Principal Molecule or Intervention	Main Finding	Translational Relevance
[28]	*Nemopilema nomurai* jellyfish venom hydrolysate	ACE-inhibitory peptide candidates IVGRPLANG and IGDEPRHQYL	An active fraction was purified; MS/MS identified two peptide sequences, and docking supported plausible ACE interactions.	Antihypertensive lead discovery
[29]	*Synanceia horrida* and *S. verrucosa* ichthyocrinotoxins	Crude and size-fractionated skin toxins	Both secretions impaired hookworm larval motility; a <3 kDa *S. horrida* fraction retained activity with limited cytotoxicity.	Anthelmintic lead discovery
[30]	Amphibian skin secretions from multiple species	Crude cutaneous secretions	Species-dependent cytotoxic, pro-inflammatory, and anti-inflammatory effects were observed in joint-relevant human cells.	Bioprospecting for modulators of joint inflammation
[31]	*Micrurus* spp. venoms	L-amino acid oxidase incorporated into gelatin films	Venoms and purified LAAO inhibited *Xanthomonas* and *Fusarium*; functionalized films extended strawberry shelf life.	Biocontrol and active food packaging
[32]	*Bothrops diporus* venom	Pooled and purified phospholipase A2 isoforms	PLA2s induced mitochondrial stress in breast cancer cells and temporally dynamic, isoform-dependent cytokine responses in PBMCs.	Tumor-bioenergetic and immunomodulatory templates
[33]	*Crotalus durissus* venom	Crotoxin and anticoagulant snake-venom PLA2s	The review integrates phospholipid hydrolysis, disruption of coagulation-complex assembly, and inflammatory modulation.	Mechanistic templates for antithrombotic development
[34]	*Vespa affinis* venom	Ves a 1 phospholipase and voxilaprevir	Molecular dynamics revealed membrane-dependent behavior and ligand-associated changes near catalytic and auxiliary regions.	Structure-guided inhibitor development
[35]	Hymenoptera venoms	Five-year venom immunotherapy	No severe systemic reactions occurred after accidental re-stings; long-term immunological changes were also documented.	Prevention of severe sting allergy
[36]	Venoms from five medically important Kenyan snakes	Four commercially available antivenoms	Three products showed broad neutralizing activity, whereas one had low binding and inadequate neutralization against several venoms.	Regional quality control, regulation, and procurement
[37]	Three viper and three elapid venoms	ECIS with varespladib, marimastat, and coagulation testing	Real-time impedance monitoring differentiated cellular injury and endpoint-specific inhibitor performance.	Quantitative preclinical screening and reduction of animal use

Abbreviations: ACE, angiotensin-converting enzyme; ECIS, electrical cell-substrate impedance sensing; LAAO, L-amino acid oxidase; PBMCs, peripheral blood mononuclear cells; PLA2, phospholipase A2.
